# Angiotensin II in experimental hyperdynamic sepsis

**DOI:** 10.1186/cc8185

**Published:** 2009-11-30

**Authors:** Li Wan, Christoph Langenberg, Rinaldo Bellomo, Clive N May

**Affiliations:** 1 Howard Florey Institute, University of Melbourne, Grattan Street, Parkville, Melbourne, Victoria 3052, Australia; 2 Australian and New Zealand Intensive Care Research Centre, Department of Epidemiology and Preventive Medicine, Monash University, Burnett Building, Commercial Road, Prahran, Melbourne, Victoria, Australia; 3 Department of Intensive Care and Department of Medicine, Austin Health, Studley Road, Heidelberg, Melbourne Victoria 3084, Australia; 4 Department of Pharmacology, University of Melbourne, Grattan Street, Parkville, Melbourne, Victoria 3052, Australia

## Abstract

**Introduction:**

Angiotensin II (Ang II) is a potential vasopressor treatment for hypotensive hyperdynamic sepsis. However, unlike other vasopressors, its systemic, regional blood flow and renal functional effects in hypotensive hyperdynamic sepsis have not been investigated.

**Methods:**

We performed an experimental randomised placebo-controlled animal study. We induced hyperdynamic sepsis by the intravenous administration of live E. coli in conscious ewes after chronic instrumentation with flow probes around the aorta and the renal, mesenteric, coronary and iliac arteries. We allocated animals to either placebo or angiotensin II infusion titrated to maintain baseline blood pressure.

**Results:**

Hyperdynamic sepsis was associated with increased renal blood flow (from 292 +/- 61 to 397 +/- 74 ml/min), oliguria and a decrease in creatinine clearance (from 88.7 +/- 19.6 to 47.7 +/- 21.0 ml/min, *P* < 0.0001). Compared to placebo, Ang II infusion restored arterial pressure but reduced renal blood flow (from 359 +/- 81 ml/min to 279 +/- 86 ml/min; *P* < 0.0001). However, despite the reduction in renal blood flow, Ang II increased urine output approximately 7-fold (364 +/- 272 ml/h vs. 48 +/- 18 ml/h; *P* < 0.0001), and creatinine clearance by 70% (to 80.6 +/- 20.7 ml/min vs.46.0 +/- 26 ml/min; *P* < 0.0001). There were no major effects of Ang II on other regional blood flows.

**Conclusions:**

In early experimental hypotensive hyperdynamic sepsis, intravenous angiotensin II infusion decreased renal blood while inducing a marked increase in urine output and normalizing creatinine clearance.

## Introduction

Acute kidney injury (AKI) affects 5 to 7% of all hospitalized patients [1] and independently increases mortality and the cost and complexity of care. Sepsis is the leading cause of ARF in the intensive care unit and septic ARF occurs most commonly in critically ill patients [2]. The mortality of septic ARF remains high despite the application of renal replacement therapies and other supportive treatments [1,3].

The cardiovascular hallmark of severe sepsis is hypotension, which is widely held to cause reduced renal blood flow (RBF) leading to AKI [4-6]. However, in recent animal studies reproducing the hyperdynamic sepsis typically seen in critically ill humans, RBF was found to be increased [7,8]. In this setting, despite renal vasodilatation and a marked increase in RBF, animals still developed oliguria and decreased creatinine clearance [9]. These findings concur with the limited studies of RBF in human sepsis [10,11]. They suggest that afferent and even greater efferent arteriolar vasodilatation may occur in early hyperdynamic sepsis. If this were true, selective vasoconstriction of the efferent arteriole with angiotensin II (Ang II) in this setting may be physiologically logical and safe and may attenuate renal dysfunction.

Ang II is a powerful vasoconstrictor hormone that causes a preferential increase in efferent arteriolar resistance [12]. It has been rarely used in hyperdynamic sepsis [13,14] due to concerns about its possible deleterious effects on regional blood flow and renal function. However, there are no studies of its effects on regional blood flows when administered by continuous infusion to restore blood pressure in hyperdynamic hypotensive sepsis. More relevant, there are no studies to confirm whether its potential adverse effects on renal blood flow are functionally important. To address these limitations in our knowledge and given the rationale that selective efferent arteriolar vasoconstriction may be desirable in this setting, we conducted a randomized controlled animal study and measured the systemic and regional hemodynamic effects and the renal functional effects of Ang II infusion compared with placebo in an animal model of hypotensive hyperdynamic sepsis.

## Materials and methods

### Animal preparation

Experiments were completed on eight adult Merino ewes (weighing 35 to 50 kg), housed in individual metabolic cages. Experimental procedures were approved by the Animal Experimentation Ethics Committee of the Howard Florey Institute, Melbourne, Australia, under guidelines laid down by the National Health and Medical Research Council of Australia.

Prior to the studies, sheep underwent three aseptic surgical procedures, each separated by two weeks. Anesthesia was induced with intravenous sodium thiopental (15 mg/kg) and, following intubation, it was maintained with 1.5 to 2.0% isoflurane/oxygen. In the first stage, sheep were prepared with bilateral carotid arterial loops. In two further operations, sheep were implanted with flow probes as previously described [7,8]. Briefly, transit-time flow probes (Transonic Systems Inc., Ithaca, NY, USA) were implanted on the ascending aorta (20 mm) and the left circumflex coronary artery (3 mm). Two weeks later, they were inserted around the mesenteric artery (6 mm), the left renal artery (4 mm), and the left external iliac artery (6 mm). Antibiotics (0.4 g procaine benzyl penicillin, 0.5 g dihydrostreptomycin sulphate (Norbrook Laboratories, UK) were administered prophylactically for three days post-surgery. Post-surgical analgesia was maintained with intramuscular injection of flunixin meglumine (1 mg/kg; Mavlab, Brisbane, Australia) at the start of surgery, and then 4 and 16 hours post-surgery.

Experiments commenced at least two weeks after surgery and were conducted on conscious sheep. On the day prior to the experiments, arterial and venous cannulae were inserted as described previously [7,8]. Cannulae were connected to pressure transducers (CDX III. Cobe, Denver, CO, USA) tied to the wool on the sheeps' backs. Pressures were adjusted to account for the height of the transducers above the heart. A bladder catheter was inserted for urine collection.

Data from the flow probes were collected via flow-meters (Transonic Systems Inc., Ithaca, NY, USA). The use of chronically implanted transit-time flow probes for the accurate measurement of regional blood flow has been described previously [15,16]. Analog signals for mean arterial pressure (MAP), central venous pressure (CVP), cardiac output (CO) and RBFs were collected at 100 Hz for 10 seconds at 1 minute intervals throughout the experiment on a computer using custom written software. For the figures, data were grouped into means values for every 15 minutes.

### Experimental protocol

Baseline measurements were collected for a 120 minute control period before the induction of sepsis by intravenous injection of live *Escherichia coli *(3 × 10^9 ^colony forming units) over five minutes at 01.00 AM. Approximately 8 to 12 hours after the initial bolus, animals typically reached the pre-defined cardiovascular criteria for randomization (hyperdynamic sepsis): 10% decrease in MAP, 50% increase in heart rate and 30% increase in CO.

After reaching the criteria for septic shock, animals were observed for a 120 minute pre-treatment period before being randomly assigned to receive a six-hour intravenous infusion of either Ang II (55 ± 78 ng/kg/min, range 4.25 to 450 ng/kg/min) or vehicle (saline). The dose of Ang II was titrated to maintain MAP at the pre-sepsis control level, with two of the sheep requiring increases in infusion rate during the treatment period to maintain MAP. Blood samples were taken the day before the induction of sepsis, at the end of the sepsis control period, and every two hours during the six-hour treatment period. Urine was collected and sampled every two hours throughout the experiment from the bladder catheter using an automated fraction collector. The creatinine clearance (Creatinine_Urine_/Creatinine_Plasma _× Urine_Volume_/time) and fractional excretion of sodium (Sodium_Urine_/Sodium_Plasma _× Creatinine_Plasma_/Creatinine_Urine _× 100) were calculated. At the end of the experiment, all catheters were removed and animals were allowed to recover for 10 to 14 days before being crossed over to the other arm of the study.

### Statistical analysis

Data are presented as means with standard deviations and all comparisons were performed by repeated measures analysis of variance [17]. All analyses were performed using SAS version 9.1 (SAS Institute, Cary, NC, USA). To correct for the effect of multiple comparisons, only a two-sided *P *< 0.01 was considered statistically significant.

## Results

### Effects of sepsis

After administration of *E. coli*, all animals developed features of sepsis: (temperature of >41°C, a respiratory rate >40 breaths per minute, use of accessory muscles of respiration, hypotension, tachycardia, lassitude, anorexia). Two sheep died following induction of sepsis.

**Figure 1:**
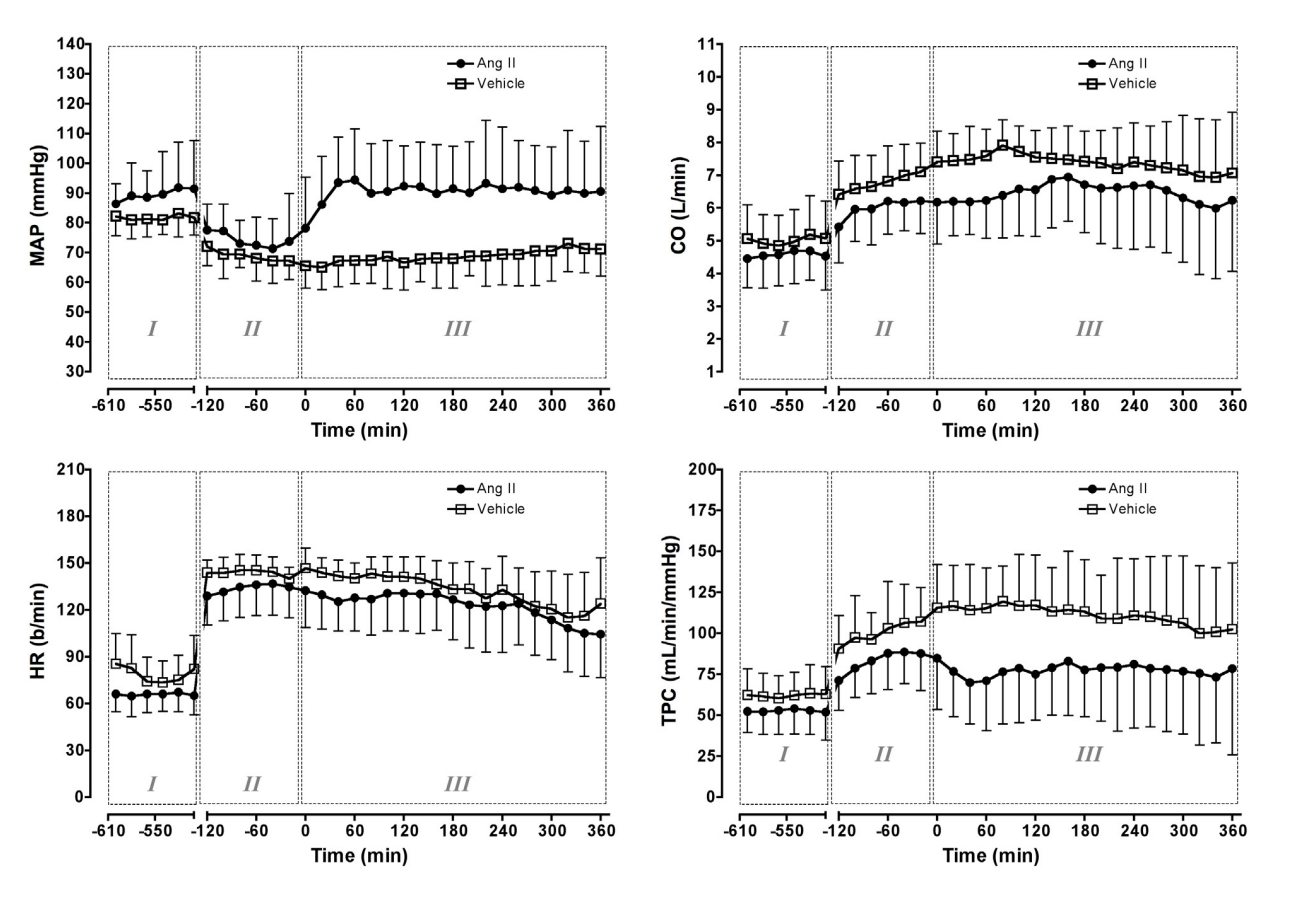
Effect of intravenous angiotensin II or vehicle on systemic hemodynamics. Phase I = control period, two hours before *Escherichia coli *administration; Phase II = sepsis control period two hours before treatment; Phase III = six hours of treatment with angiotensin (Ang) II or vehicle. CO = cardiac output; HR = heart rate; MAP = mean arterial pressure; TPC = total peripheral conductance. Means (standard deviation), n = 6.

The onset of severe sepsis was associated with peripheral vasodilatation, hypotension and an increase in CO (hyperdynamic septic state). MAP decreased (from 86.3 ± 7.2 to 71.8 ± 7.2 mmHg, *P *< 0.0001) and total peripheral conductance increased (*P *< 0.0001; Figure 1). These changes were accompanied by increases in CO and heart rate (Figure 1) and a reduction in stroke volume (67.5 ± 11.6 to 46.4 ± 7.5 ml/beats, *P *< 0.0001).

**Figure 2:**
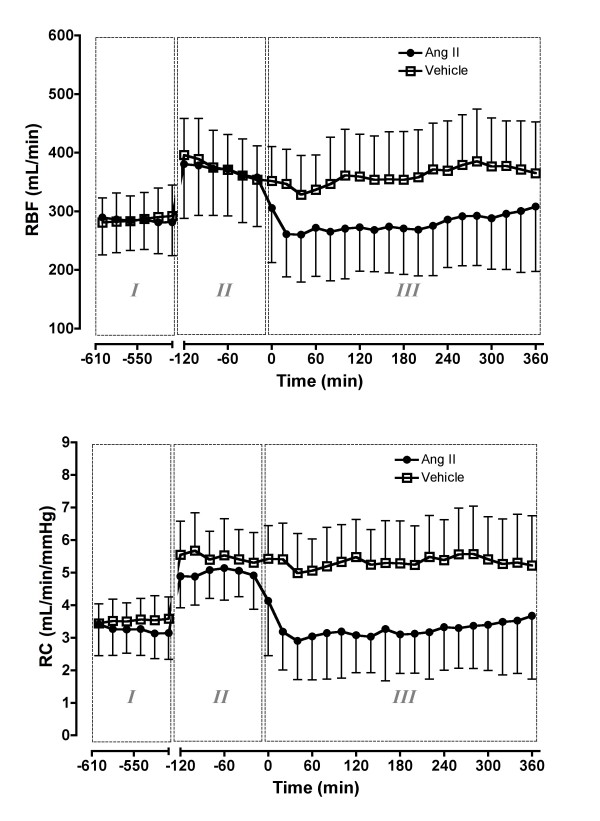
Effect of intravenous angiotensin II or vehicle on renal blood flow (RBF) and renal conductance (RC). Phase I = control period, two hours before *Escherichia coli *administration; Phase II = sepsis control period, two hours before treatment; Phase III = six hours of treatment with angiotensin (Ang) II or vehicle. Means (standard deviation), n = 6.

**Figure 3:**
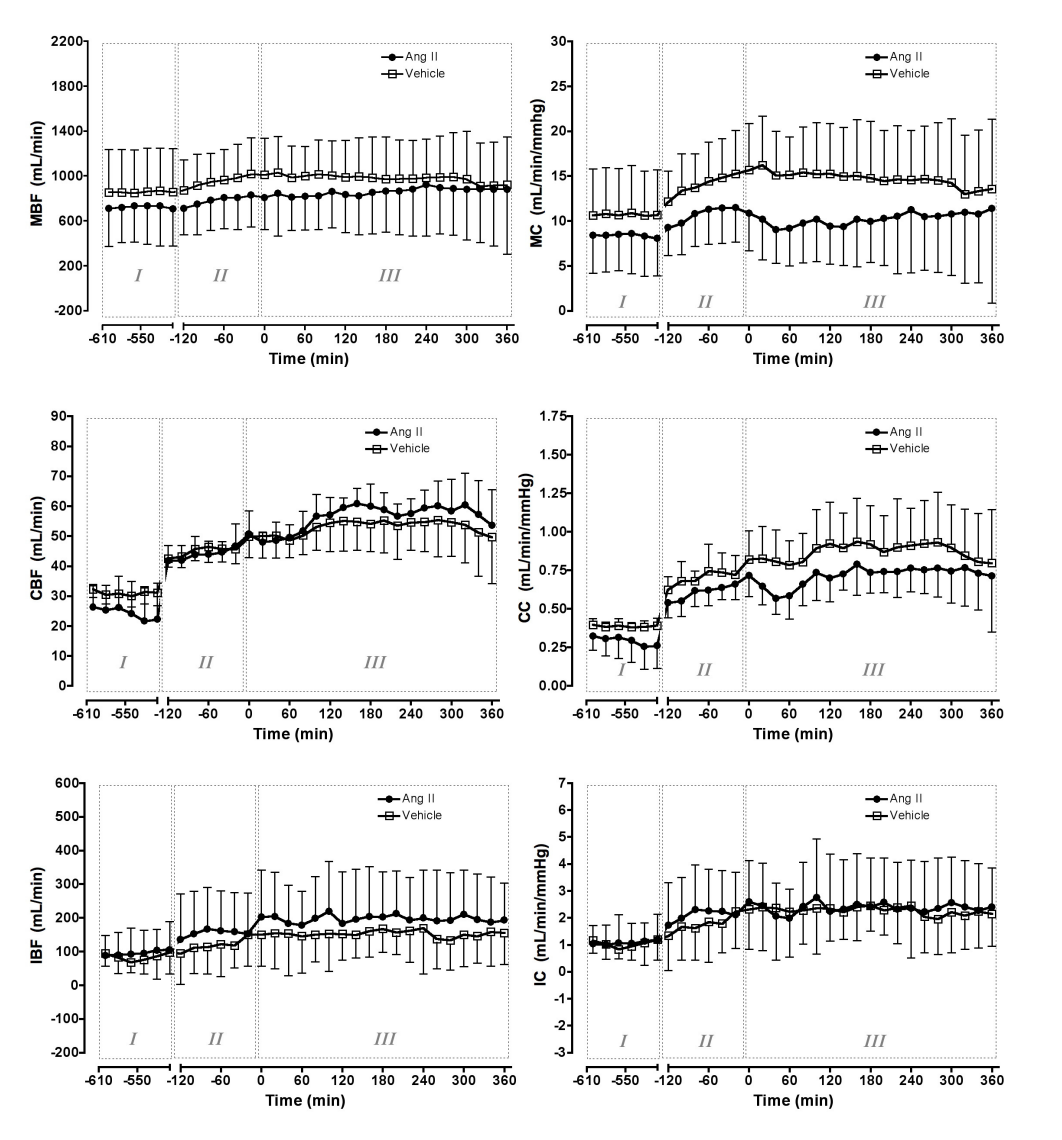
Effect of intravenous angiotensin II or vehicle on regional haemodynamics. Phase I = control period, two hours before *Escherichia coli *administration; Phase II = sepsis control period, two hours before treatment; Phase III = six hours of treatment with angiotensin (Ang) II or vehicle. IBF = iliac blood flow; IC = iliac conductance; MBF = mesenteric blood flow; MC = mesenteric conductance; Means (standard deviation), n = 6.

Sepsis caused pronounced vasodilatation in all regional vascular beds with increases in renal conductance (3.4 ± 0.8 to 5.2 ± 0.9 ml/min/mmHg, *P *< 0.0001) and RBF (292.3 ± 60.5 to 396.6 ± 74.1 ml/min, *P *< 0.0001; Figure 2). There were similarly large increases in coronary conductance and blood flow and in iliac conductance and blood flow (Figure 3). Mesenteric conductance and mesenteric blood flow also increased (Figure 3).

**Figure 4:**
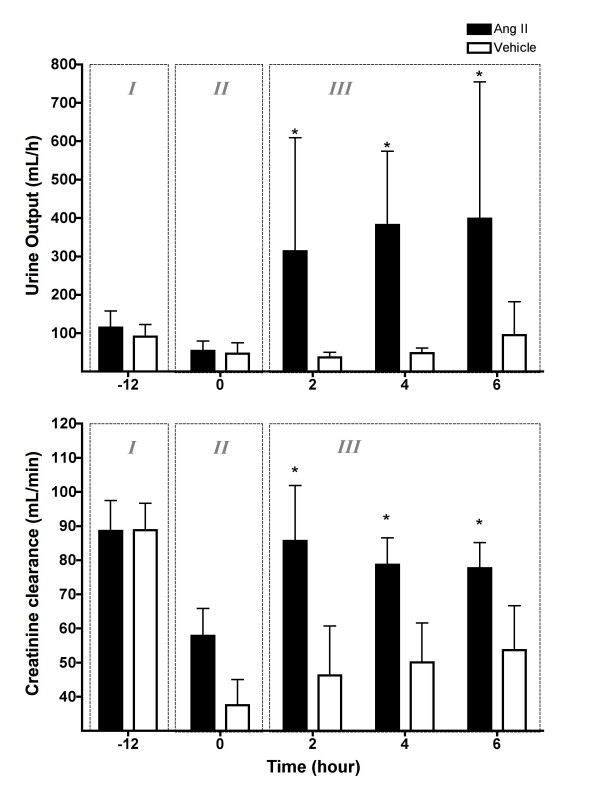
Effect of intravenous angiotensin II or vehicle on urine output and creatinine clearance. Phase I = control period, two hours before *Escherichia coli *administration; Phase II = sepsis control period, two hours before treatment; Phase III = six hours of treatment or vehicle. Means (standard deviation), n = 6, * *P *< 0.05 comparison between treatment and vehicle. Ang = angiotensin.

Despite the increase in RBF during sepsis, there was a 46% decrease in urine output (102.6 ± 38.1 to 50.5 ± 25.4 ml/h, *P *< 0.01) and a 43% decrease in creatinine clearance (88.7 ± 19.6 to 47.7 ± 21.0 ml/min, *P *< 0.01; Figure 4). Fractional excretion of sodium (FENa), however, did not change (0.47 ± 0.35 to 0.45 ± 0.35%, *P *> 0.05).

Sepsis also caused a significant decrease in partial pressure of arterial oxygen and an increase in blood lactate but no other biochemical changes (Table 1).

**Table 1:** Blood and plasma levels of biochemical variables during the pre-sepsis period, immediately before treatment, and then at 2, 4 and 6 hours of Ang II or vehicle infusion (n = 6 in both groups)

Variables	Treatment	Pre-sepsis	0 h	2 h	4 h	6 h
**Ph**	Ang II	7.489 ± 0.025	7.518 ± 0.036	7.509 ± 0.049	7.518 ± 0.055	7.517 ± 0.060
	Vehicle	7.449 ± 0.075	7.496 ± 0.054	7.520 ± 0.055	7.506 ± 0.049	7.496 ± 0.042
**PaCO_2_ (Torr (kPa))**	Ang II	34.9 ± 1.9 (4.65 ± 0.25)	31.0 ± 3.4* (4.13 ± 0.45)	34.5 ± 4.0 (4.60 ± 0.53)	35.2 ± 3.8 (4.69 ± 0.51)	36.0 ± 3.6 (4.80 ± 0.48)
	Vehicle	33.8 ± 2.3 (4.37 ± 0.31)	31.6 ± 2.3* (4.21 ± 0.31)	36.0 ± 6.3 (4.80 ± 0.84)	36.0 ± 6.8 (4.80 ± 0.91)	37.3 ± 10.3 (4.97 ± 1.37)
**Pa O_2_ (Torr (kPa))**	Ang II	97.5 ± 12.0 (13.0 ± 1.6)	85.5 ± 3.5* (11.4 ± 0.5)	89.0 ± 6.8 (11.9 ± 0.9)	87.6 ± 10.3 (11.7 ± 1.4)	84.9 ± 10.0 (11.3 ± 1.3)
	Vehicle	97.8 ± 5.1 (13.0 ± 0.7)	92.3 ± 10.2* (12.4 ± 1.4)	83.4 ± 10.4 (11.1 ± 1.4)	89.3 ± 7.9 (11.9 ± 1.1)	83.1 ± 9.1 (11.1 ± 1.2)
**HCO_3_^-^ (mmol/l)**	Ang II	26.3 ± 1.6	24.9 ± 1.5	27.2 ± 2.4	28.4 ± 2.9	29.0 ± 2.8
	Vehicle	23.3 ± 3.7	24.3 ± 2.3	29.2 ± 5.1	28.4 ± 6.9	28.7 ± 8.8
**BE (mmol/l)**	Ang II	3.4 ± 1.6	2.7 ± 1.4	4.4 ± 2.6	5.5 ± 3.0	5.9 ± 3.1
	Vehicle	0.0 ± 4.6	1.6 ± 2.7	6.2 ± 4.7	5.2 ± 6.1	5.3 ± 7.5
**K^+^ (mmol/l)**	Ang II	4.3 ± 0.2	4.1 ± 0.4	4.1 ± 0.6	4.0 ± 0.7	3.9 ± 0.7
	Vehicle	4.0 ± 0.4	3.9 ± 0.3	4.1 ± 0.3	4.3 ± 0.4	4.2 ± 0.4
**Na^+^ (mmol/l)**	Ang II	142.3 ± 3.8	143.3 ± 3.2	143.2 ± 2.5	142.0 ± 2.5	141.3 ± 2.4
	Vehicle	143.2 ± 1.9	143.8 ± 3.4	144.0 ± 4.0	144.0 ± 4.6	144.0 ± 4.6
**Ca^2+^ (mmol/l)**	Ang II	1.2 ± 0.1	1.1 ± 0.1*	1.1 ± 0.1	1.0 ± 0.1	1.0 ± 0.2
	Vehicle	1.2 ± 0.1	1.1 ± 0.1*	1.1 ± 0.1	1.1 ± 0.0	1.2 ± 0.0
**Cl^-^ (mmol/l)**	Ang II	109.7 ± 3.3	111.5 ± 5.9	106.8 ± 3.4	105.0 ± 3.7	103.3 ± 4.1
	Vehicle	108.8 ± 2.1	108.0 ± 3.2	108.0 ± 3.6	107.8 ± 4.3	108.5 ± 4.2
**Lactate (mmol/l)**	Ang II	0.6 ± 0.2	2.1 ± 1.4*	2.1 ± 1.6	1.8 ± 1.2	1.7 ± 1.4
	Vehicle	0.5 ± 0.1	1.9 ± 1.2*	1.8 ± 1.3	1.5 ± 1.3	1.4 ± 1.4
**HB (g/dl)**	Ang II	9.9 ± 1.2	10.8 ± 1.0	10.1 ± 1.3	9.8 ± 0.9	9.9 ± 1.1
	Vehicle	9.7 ± 1.6	10.2 ± 1.1	9.9 ± 1.1	9.6 ± 1.1	9.6 ± 1.0

Results are mean ± standard deviation. There were no significant differences between the Ang II and vehicle groups. * statistical difference between pre-sepsis period and sepsis control period. Ang II = angiotensin II; BE = base excess; HB = hemoglobin; PaCO_2 _= partial pressure of arterial carbon dioxide; PaO_2 _= partial pressure of arterial oxygen.

### Effects of infusion of angiotensin II during sepsis

#### Systemic hemodynamics

Intravenous infusion of Ang II increased and maintained MAP at baseline levels, while animals assigned to receive vehicle remained hypotensive (Figure 1). The Ang II-induced increase in arterial pressure resulted from peripheral vasoconstriction with a small reduction in CO, but no significant effect on heart rate (Figure 1).

#### Regional hemodynamics

Ang II infusion significantly reduced renal conductance and RBF to 3.3 ± 1.4 ml/min/mmHg and 278.8 ± 86.0 ml/min (both *P *< 0.0001), respectively, which then returned to levels similar to those in the pre-sepsis period (3.4 ± 0.8 ml/min/mmHg and 292.3 ± 60.5 ml/min, respectively, both *P *> 0.05; Figure 2). These effects were maintained for the six hour infusion, while in the vehicle group, renal conductance (5.2 ± 1.3 ml/min/mmHg) and RBF (358.7 ± 80.8 mL/min) remained elevated (Figure 2). Ang II had a significant but less potent vasoconstrictor effect on other vascular beds (Figure 3).

#### Renal function

Compared with vehicle infusion, Ang II infusion increased urine output more than seven-fold (364.3 ± 272.1 ml/h vs. 48.1 ± 18.1 mL/h; *P *< 0.0001; Figure 4). This effect was maintained throughout the experiment. Ang II infusion also increased creatinine clearance to 80.6 ± 20.7 ml/min, a value similar to pre-sepsis levels (88.7 ± 19.6 ml/min, *P *> 0.05), while, in the vehicle-treated group, creatinine clearance remained low (46.0 ± 26.0 mL/min; *P *< 0.0001; Figure 4).

#### Respiratory and acid base changes

Infusion of Ang II had no significant effects on arterial blood gases, plasma electrolytes or acid base variables, compared with vehicle (Table 1). However, Ang II significantly increased FENa (0.66 ± 0.23 to 2.71 ± 2.29%, *P *< 0.0001).

## Discussion

In a model of hypotensive hyperdynamic sepsis, we examined the systemic and regional hemodynamic effects and renal functional effects of intravenous Ang II infusion. We found that Ang II at a dose titrated to restore MAP to baseline levels induced systemic vasoconstriction with limited vasoconstrictive effects on the mesenteric but not coronary or iliac vascular beds. We found, however, that Ang II decreased RBF and renal conductance to pre-sepsis levels, while increasing urine output, creatinine clearance and fractional natriuresis.

One of the characteristics of severe hypotensive hyperdynamic sepsis is peripheral vasodilatation [18,19]. However, it has been suggested that, in sepsis, such generalized vasodilatation might spare the kidney such that RBF decreases due to vasoconstriction, ischemia develops and renal function declines [4-6]. In contrast, in our model of hyperdynamic sepsis, we found intense renal vasodilatation and increased RBF, as previously reported [8,9]. Despite renal hyperemia, there was a significant decline in renal function, as shown by the decreased urine output and creatinine clearance. These changes suggest a marked reduction in glomerular filtration rate (GFR) and its major determinant, intra-glomerular capillary pressure. The effect of Ang II on urine output, creatinine clearance and natriuresis occurring in the setting of renal vasoconstriction and decreased RBF, as seen in our study, is consistent with the hypothesis that Ang II causes an increase in glomerular filtration pressure, possibly through selective efferent arteriolar vasoconstriction or changes in mesangial cell tone or both. To our knowledge, this is the first report of such effects in hyperdynamic sepsis. Previous animal studies in rats demonstrated a likely role for Ang II to oppose the hypotensive response to lipopolysaccharide-induced sepsis [20] and a reduced systemic pressor response to Ang II boluses at doses similar to ours, an effect which was associated with a variable renal vasoconstrictor response [21]. None of these studies, however, measured the renal functional effect or systemic and renal hemodynamic effect of extended Ang II infusion.

The increase in urinary output, fractional natriuresis and creatinine clearance induced by infusion of Ang II in sepsis seems unlikely to be simply secondary to increases in arterial pressure. First, the MAP value during Ang II infusion was similar to baseline values and yet urine output and fractional natriuresis were much greater. Second, in previous identical studies, norepinephrine and epinephrine infusion caused similar increases in arterial pressure but produced much smaller and more transient improvements in renal function than Ang II [7,22].

The renal effect of Ang II may relate to its selective effects on intrarenal hemodynamics, where it controls tone in the afferent and efferent arterioles [14] and mesangial cells [23]. Infusion of Ang II increases filtration fraction [24-26], which has been proposed to result from a greater sensitivity of the efferent than the afferent arterioles to its vasoconstrictor action, a notion supported by several studies [27-32]. In contrast, the afferent and efferent arterioles have been shown to respond similarly to norepinephrine [27].

If Ang II is to be used in human sepsis, it is important to assess whether this treatment has harmful effects. We found that Ang II caused only limited reductions in blood flow to the heart, gut or skeletal muscle. Additionally, we found no evidence of adverse effects on electrolyte or lactate levels. Desensitization to the effects of vasoconstrictor agents, including Ang II, in sepsis is well established. In this study, the levels of Ang II required to increase MAP by 20 mmHg were up to five-fold more than the dose required in healthy animals [33]. A similar desensitization to Ang II may occur in septic patients compared with healthy humans [12,34]. This phenomenon is not fully understood, but may result from high levels of nitric oxide counteracting the vasoconstrictor effect of Ang II or from down regulation of angiotensin AT-_1 _receptors [35].

Our study has both strengths and limitations. It is randomized and placebo-controlled, conducted in conscious animals to remove the confounding effects of sedation or anaesthesia. Blood flows were measured by highly accurate probes inserted several weeks before the experiment. Furthermore, the renal effects of Ang II are clear, internally consistent and kidney specific. On the other hand, the indirect measurement of GFR by means of creatinine clearance is of limited accuracy in the absence of a steady state. The changes we observed, however, were marked and strongly suggestive of a true effect. Our model does not completely reproduce severe human sepsis. However, the systemic inflammatory syndrome developed and three major criteria for a hyperdynamic circulation were present throughout the study period. The decrease in arterial pressure and urine output and the increase in lactate meant that our animals fulfilled the ACCP/SCCM consensus criteria for severe sepsis [36]. The mortality rate of 25% seen in our animals is similar to the 30% mortality rate seen in severe sepsis in humans. Nonetheless, the septic state in our animals was not sustained beyond 8 to 12 hours and our observations may not apply to prolonged or recurrent sepsis as seen in other large animal models of sepsis [37]. In addition, our animals, unlike many septic humans, did not have old age, vascular disease, hypertension or diabetes. These differences between our model and human sepsis must be taken into account in the interpretation of our findings. We did not measure Ang II levels thus making it impossible to compare Ang II levels during the natural response to sepsis with those achieved duringAng II infusion. We did not administer fluid resuscitation, although such resuscitation is typically performed in human sepsis and might have modified our findings. The CO and total peripheral conductance during the untreated septic state in placebo animals showed small differences from animals allocated to Ang II. These differences may have affected our findings. Finally, we acknowledge that increases in blood pressure can, independent of the drug used, induce a diuresis [38]. However, the effects fo Ang II or renal function during sepsis were more potent than those of doses of epinephrine and norepinephrine that caused similar increases in arterial pressure [7,22]. It is also possible that the combination of lower dose vasopressor drugs (multimodal therapy) would achieve better renal protection with lower systemic side effects.

## Conclusions

We found that, in a large animal model of experimental hypotensive hyperdynamic sepsis, infusion of Ang II at a dose that restores MAP to pre-sepsis levels, significantly reduced RBF and simultaneously increased urine output, fractional natriuresis and creatinine clearance. Ang II caused these renal changes without major adverse effects on blood flows to other vital organs, blood lactate or biochemical variables. These findings justify further investigations of Ang II in experimental and human sepsis.

## Key messages

• Experimental hyperdynamic hypotensive sepsis can be associated with renal vasodilatation and hyperemia while renal function becomes impaired

• The combination of renal vasodilatation with decreased GFR suggests combined afferent and efferent arteriolar vasodilatation with greater arteriolar dilatation

• Ang II infusion restores renal hemodynamic to normal

• Ang II-induced restoration of intra-renal hemodynamics to normal is associated with improved creatinine clearance and a marked increase in urine output.

## Abbreviations

AKI: acute kidney injury; Ang II: angiotensin II; CO: cardiac output; CVP: central venous pressure; FENa: fractional excretion of sodium; GFR: glomerular filtration rate; MAP: mean arterial pressure; RBF: renal blood flow.

## Competing interests

As a result of this study, Drs. Clive May and Rinaldo Bellomo have applied for patent protection for the use of angiotensin II to treat septic acute kidney injury in man.

## Authors' contributions

CNM, LW, CL and RB designed the study. LW and CNM performed the experiments and data analysis. All authors participated in the drafting of the final manuscript. All authors read and approved the final manuscript.
